# No-touch endoscopic full-thickness resection using reopenable-clip over-the-line method for gastric gastrointestinal stromal tumor

**DOI:** 10.1055/a-2262-8203

**Published:** 2024-03-01

**Authors:** Hitoshi Mori, Noriya Uedo, Satoki Shichijo

**Affiliations:** 1 Department of Gastrointestinal Oncology, Osaka International Cancer Institute, Osaka, Japan

With advancement of endoscopic resection and wound closure techniques, endoscopic resection of gastric gastrointestinal stromal tumors (GISTs) has evolved rapidly in recent years. However, concerns remain regarding the oncologic safety of this procedure, particularly the risk of tumor injury and the adequacy of the resection margins. To solve these problems, the no-touch endoscopic full-thickness resection (EFTR) technique, in which the tumor is removed with a 0.5–1.0-cm resection margin of the muscularis propria, has recently been proposed 1 .

A woman aged in her 40s was diagnosed with a submucosal tumor on the upper gastric corpus ( Fig. 1 **a** ). The lesion increased in size and led to histological diagnosis of GIST and referral to our hospital for endoscopic treatment. No-touch EFTR was performed using a therapeutic videoendoscope (GIF-H290T; Olympus Medical Systems Co. Ltd., Tokyo, Japan) with transparent hood (D-201-11804; Olympus), Flush Knife BT 2.0 (Fujifilm Medica Co. Ltd., Tokyo, Japan), and ITknife2 (Olympus). The procedure consisted of the following steps: (i) marking around the line skirting the submucosal tumor protrusion; (ii) circumferential mucosal incision outside the markings without submucosal injection; (iii) creation of the deep mucosal/submucosal groove to the surface of the muscularis propria; (iv) application of clip-and-line traction (3–0 polyester suture line) to the anal side of the lesion; (v) muscle incision from the anal side along the mucosal/submucosal groove ( Fig. 1 **b** ); (vi) defect closure with reopenable-clip over-the-line method using 4–0 nylon suture line ( Fig. 1 **c** ) 2 ; and (vii) lesion retrieval ( Fig. 1 **d** , Video 1 ). The total procedure time was 75 minutes.

**Fig. 1:**
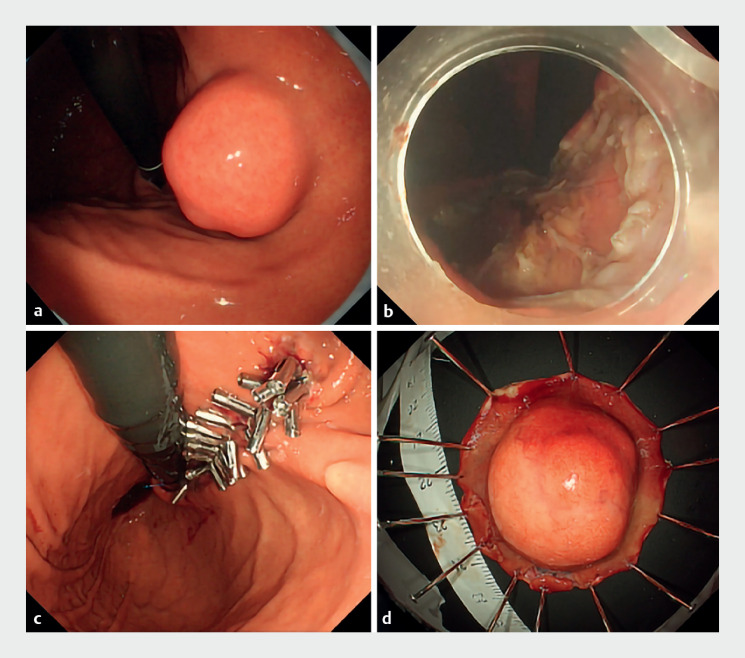
Tumor resection and closure. **a** Endoscopic appearance of the lesion. **b** Full-thickness defect after no-touch endoscopic full-thickness resection. **c** Wound closure by the reopenable-clip over-the-line method. **d** The resected specimen.

No-touch endoscopic full-thickness resection using reopenable-clip over-the-line method for gastric gastrointestinal stromal tumor.Video 1

The patient was discharged uneventfully. Histological examination confirmed low risk GIST with free resection margin ( Fig. 2 ).

**Fig. 2:**
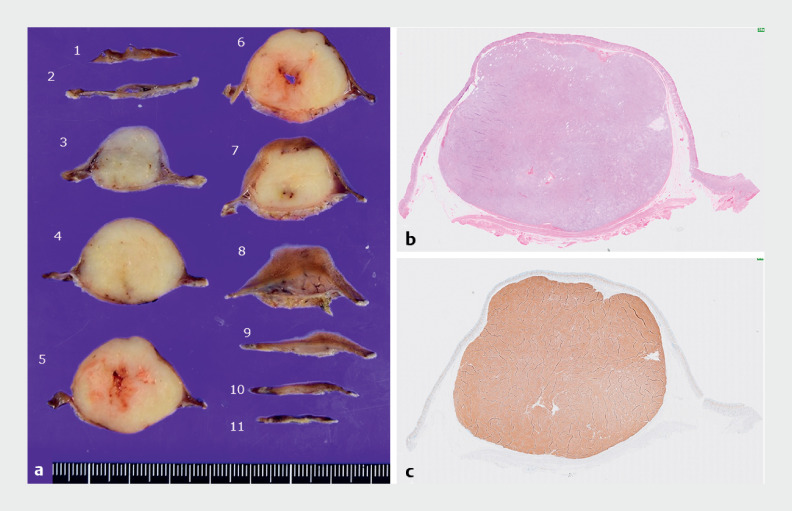
Histological findings of the lesion. **a** Sectioned specimen. **b** Hematoxylin and eosin staining. **c** Positive immunostaining with c-kit.

Endoscopy_UCTN_Code_TTT_1AO_2AG

## Notes

Endoscopy E-Videos https://eref.thieme.de/e-videos
*E-Videos* is an open access online section of the journal *Endoscopy* , reporting on interesting cases and new techniques in gastroenterological endoscopy. All papers include a high-quality video and are published with a Creative Commons CC-BY license. Endoscopy E-Videos qualify for HINARI discounts and waivers and eligibility is automatically checked during the submission process. We grant 100% waivers to articles whose corresponding authors are based in Group A countries and 50% waivers to those who are based in Group B countries as classified by Research4Life (see: https://www.research4life.org/access/eligibility/ ). This section has its own submission website at https://mc.manuscriptcentral.com/e-videos .
